# Patterns, aetiology and risk factors of intimate partner violence-related injuries to head, neck and face in Chinese women

**DOI:** 10.1186/1472-6874-14-6

**Published:** 2014-01-10

**Authors:** Janet Yuen-Ha Wong, Anna Wai-Man Choi, Daniel Yee-Tak Fong, John Kit-Shing Wong, Chu-Leung Lau, Chak-Wah Kam

**Affiliations:** 1 School of Nursing, Li Ka Shing Faculty of Medicine, The University of Hong Kong, 4/F, William M.W. Mong Block, 21 Sassoon Road, Pokfulam, Hong Kong SAR, The People's Republic of China; 2 Department of Social Work and Social Administration, Faculty of Social Science, The Centennial Campus, The University of Hong Kong, Room 534, Jockey Club Tower, Pokfulam, Hong Kong SAR, The People's Republic of China; 3 Department of Accident and Emergency Medicine, Tuen Mun Hospital, Tuen Mun, Hong Kong SAR, The People's Republic of China; 4 Department of Accident and Emergency Medicine, Pok Oi Hospital, Yuen Long, Hong Kong SAR, The People's Republic of China

**Keywords:** Head injuries, Maxillofacial injuries, Intimate partner violence, Abused Chinese women

## Abstract

**Background:**

Intimate partner violence (IPV) related injuries have been recognized among health care professionals. However, few studies have provided detailed information on injuries to the head, neck and face regions in Chinese women. As abused Chinese women are generally unwilling to disclose IPV and there are differences in socio-demographic characteristics, societal norms and behaviours, the women may exhibit different patterns, aetiology and risk factors of IPV-related HNF injuries. This study aims to examine the patterns of head, neck and face injuries presenting to Accident and Emergency departments, including the anatomical regions, types, severity, aetiology and demographic and non-demographic risk factors of injuries inflicted by intimate partners in Chinese context.

**Methods:**

Medical charts of 223 women presented to the Accident and Emergency departments of two regional hospitals in Hong Kong between January 2010 and December 2011 were reviewed independently by two reviewers.

**Results:**

Head, neck and face injuries remained the most common injuries found in abused Chinese women (77.6%), and punching with a fist was the most common aetiology (60.2%). In particular, punching with a fist was significantly associated on the upper third of the maxillofacial region (p = .01) and the back part of the head (p = .03). Moreover, cohabiting and separated women were more likely to have multiple injuries than those who were married (OR = 3.3, 95% CI = 1.4, 7.8; OR = 2.1, 95% CI = .4, 11.9).

**Conclusions:**

The findings enhance the understanding of head, neck and face injuries and inform clinicians about the linkage among injuries and risks in abused Chinese women.

## Background

Intimate partner violence (IPV) refers to acts of physical, sexual or emotional abuse by a current or former intimate partner, whether cohabiting or not [1]. It has been recognized as a public health issue by the World Health Organization since 2002 [2,3]. Furthermore, it has contributed to the high cost of health care utilization with evidence that health care cost of abused women ranged from 1.6 to 2.3-fold increase compared to non-abused women in the Unites States [4]. Of IPV-related physical injuries, around 40% have been found to involve the head, neck or face (HNF) regions [5-8]. Although most HNF injuries are soft tissue and cutaneous injuries, severe injuries can also occur; for example, being slapped in the face could result in a perforated tympanic membrane [9], being banged against a wall could result in microstructural shearing tears to the brain tissue [10], being choked could result in asphyxia [11] and substantial neurological impairment with or without loss of consciousness, confusion or memory [12,13].

Understanding patterns of IPV-related HNF injuries is essential. For example, a study found that the frontal lobe of the brain was one of the most affected anatomical sites in abused women with HNF injuries [14]. Prefrontal cortex, located in the frontal part of the head, is known to be responsible for cognitive functions, such as perception, reasoning, judgment, problem solving, and decision making [15]. Therefore, it helps to explain why cognitive impairment can be possibly found in abused women.

HNF injuries have been known as a significant indicator of IPV [16]. However, there is a paucity of research reporting the patterns, aetiology and risk factors of HNF injuries in abused Chinese women. In general, abused Chinese women are unwilling to disclose IPV and use placating and normalizing as their coping strategies [17,18]. They also consider marital conflicts and IPV to be shameful to themselves and their families [19,20]. Therefore, Chinese women may exhibit different patterns, aetiology and risk factors of IPV-related HNF injuries. Moreover, according to the recent meta-analysis conducted to examine patterns of IPV-related physical injuries in women presenting to Accident and Emergency department (AED), it indicated that no study has ever involved Chinese women. In addition, there was little demographical information, especially age, socioeconomic status and race fully reported in medical chart reviews [21]. Therefore, there is a need to examine IPV-related HNF injuries in Chinese women and investigate the demographic and non-demographic risk factors of injuries.

The present study aims to examine the patterns of HNF injuries presenting to AEDs, including the anatomical regions, types, severity, aetiology and risk factors of injuries in Chinese abused women.

## Methods

### Study design

This is a retrospective study that reviewed the medical charts of IPV victims presented with injuries at the AEDs in two district hospitals in Hong Kong between January 2010 and December 2011. They together serve a population where the annual incidence of IPV cases has been consistently the highest in Hong Kong since 2009 [22]. Ethical approval of this study has been obtained from the Ethics Committee of the Hong Kong Hospital Authority (HA).

The two AEDs share the same centralized computerized systems for patient records. They are (1) the Accident and Emergency Information System (AEIS), which holds records of patients presenting at the two AEDs, and (2) the Clinical Data Analysis & Reporting System (CDARS), which keeps records of all hospitalized in-patients and out-patients. Medical records in both systems were reviewed to ensure that cases with IPV not identified at the AED be identified during hospitalization after AED visits.

### Eligibility criteria

The eligibility criteria for our chart review included women aged 18 years or above with diagnosis of IPV-related injuries during the period between January 2010 through December 2011. We anticipated to test 14 risk factors of HNF injuries, with the sample size of at least 140 by using the 1:10 ratio [23].

IPV was assessed by either self-reporting or direct questioning by the triage nurses and emergency physicians who have received comprehensive training to assess for IPV. In total, 267 eligible medical records were identified from the two computerized systems. All 267 individual medical charts were then retrieved from the Medical Records Offices of the two hospitals and manually and independently reviewed by the first and second authors (JW and AC). Six medical charts were excluded from data analysis as the women did not have IPV-related injuries but injuries associated with indecent assaults by non-intimate partners. Also, another 38 medical charts were excluded since the patients were male. Therefore, they were excluded from the data analysis. Finally, 223 eligible cases were identified from both systems of patient records and were used for analysis.

### Data abstraction

Individual medical charts were retrieved from the Medical Records Offices of the two hospitals. To minimize misinterpretation of information from medical charts, the first two authors (JW and AC) manually and independently reviewed the medical charts generated from the AEIS and CDARS. To facilitate the data abstraction process, chart abstraction sheet was designed. The region, type and aetiology of the physical injuries were extracted. Demographical data including age, race, marital status and relationship with the perpetrator were also documented from the medical charts. Moreover, the episode of the abuse, date and time of admission and injury, procedures and treatment carried out at the AED, and discharge destination were recorded. This information would be helpful for clinicians to understand the patterns of women who sought help from health care professionals at AEDs.

In order to document the details of anatomical site and type of each HNF injury, we divided the face into upper, middle and lower thirds. The upper third consists of the frontal bone, frontal sinus and supraorbital region. The middle third consists of the orbits, the lateral orbital region, nose, maxilla and zygoma. The lower third consists of the lips, oral region and mandible. For the head and skull injuries, specific types of injuries were recorded according to the anatomical sites in the vertex, parietal, temporal and occipital regions.

For the severity of injuries, we adopted Injury Severity Score (ISS) because it has been a global and validated severity scoring system [18,24]. Each injury was assigned an Abbreviated Injury Scale (AIS) score [25] and was allocated to one of six body regions - Head, Face, Chest, Abdomen, Extremities (including Pelvis) and External in calculating the ISS. The AIS rates injuries on a scale of 0 (no injury) to 6 (unsurvivable injury), whereas, the ISS score takes values from 0 to 75. ISS was the sum of the squares of the highest AIS grade in each of the three most severely injured areas. The higher the score, the greater the severity. The aetiology of the injury can be obtained from the physician’s documentation and the hospitals’ record forms, which were particularly designed for IPV assessment.

To ensure patient confidentiality, data were recorded and analysed by using study identity numbers without identification of individuals.

### Data quality control

We anticipated data misinterpretation and missing data in data abstraction from medical chart review process. Several measures were conducted to maximize data accuracy and integrity - First, a chart abstraction sheet was designed for data collection from the medical charts. The sheet was piloted on 20 medical charts before it was used. Second, two reviewers collected the data independently. Third, quality checking was done at the end of each data collection day, in which the two reviewers met and resolved discrepancies in the collected data. There were 90% of the collected data were identical between the two data abstracters and 10% of discrepancies were discussed and resolved. Fourth, the last author (CWK), who is a physician, was available on-site to resolve any issues regarding handwriting.

### Data analysis

Data analysis was undertaken with the IBM SPSS Statistics 20.0 software package (IBM Corp, Armonk, NY, USA). Descriptive statistics were used to summarize the demographics, type, region, severity and aetiology of injuries, and corresponding procedures and treatments at the AEDs. Also, chi-squared tests were used to assess for the association of regions of injury and the causes of IPV related injury.

Moreover, risk factors of IPV-related HNF injuries were investigated by a structured multiphase logistic regression analysis, with the presence of IPV-related HNF injuries as the dependent variable, and demographics and the episode and aetiology of the injury as independent variables [26]. Structured multiphase logistic regression, instead of an ordinary multiple logistic regression, was used because it avoids inappropriate adjustment of confounding variables. Firstly, the independent variables were classified into 3 groups such that variables in group 1 may affect variables in groups 2 and 3 but not vice versa, and variables in group 2 may affect those in group 3 but not vice versa. Group 1 variables were age and race. Group 2 variable was the relationship between the woman and her perpetrator, and group 3 variables were those related to physical injuries. These variables were then entered a regression model by phases. In phase 1, a stepwise logistic regression was performed on variables in group 1. In phase II, a stepwise regression was performed on variables in group 2 after adjusting group 1 variables that were significant in phase 1. In phase III, another stepwise logistic regression was performed on variables in group 3 after adjusting variables in groups 1 and 2 that were significant in their corresponding phases. The odds ratio (OR) of a variable was taken as the one obtained in the corresponding phase of the variable. The same analysis approach was used to investigate risk factors of multiple injuries involving the head as an injured region. The Hosmer and Lemeshow (H-L) test was used to assess the goodness of fit of the logistic regression models [27]. All statistical tests were 2-sided and used a 5% level of significance.

## Results

### Participant characteristics

The study sample comprised 223 women recorded as having IPV-related injuries between January 2010 and December 2011. Table 1 shows the women’s characteristics, severity of injuries and procedures or treatment carried out at the AEDs. The mean age was around 38.6 (SD = 11.1) and the majority of them were Chinese (n = 170, 76.5%). It was found that more than half of the abused women reported multiple episodes of abuse when they were admitted to AED. The overall injuries in abused women were mild, with the ISS ranging from 0 to 5.

**Table 1:** The characteristics of women (n = 223) with IPV-related injuries including demographics, severity of injuries, and corresponding procedures or treatment carried out at A&E departments

**Participant characteristics**	**n (%)**	**Mean [SD]**	**Range**
**Age**		38.6 [11.1]	17–80
**Ethnicity**			
*Chinese*	170 (76.5)		
*Nepalese*	8 (3.6)		
*Filipino*	7 (3.1)		
*Indonesian*	7 (3.1)		
*Vietnamese*	3 (1.3)		
*Thai*	1 (.4)		
*Pakistan*	1 (.4)		
*Unknown*	24 (10.8)		
**Relationship with perpetrators**			
*Married spouse*	182 (81.6)		
*Cohabitant*	33 (14.8)		
*Boyfriend but non-cohabitant*	2 (.9)		
*Ex-husband/ex-boyfriend*	6 (2.7)		
**Episode of abuse**			
*1*^*st*^	40 (17.9)		
*2*^*nd*^	33 (14.8)		
*Multiple*	118 (52.9)		
*Undisclosed*	32 (14.5)		
**Admission time**			
*Day-time*	105 (47.1)		
*Night-time*	118 (52.9)		
**Injury Severity Score (ISS)**		1.8 [.8]	0–5
**Procedures/treatment at AED**			
*Suturing*	5 (2.3)		
*Dressing*	32 (14.5)		
*Splint/Collar*	3 (1.4)		
*ECG*	2 (0.9)		
*Blood test/ Haemostix*	20 (8.6)		
*Oral medication*	23 (10.4)		
*Urine test*	14 (7)		
*Ultrasound*	4 (2)		
*X-ray*	98 (44.3)		
*CT*	12 (5.4)		
**Discharge**			
*Home*	165 (74)		
*Admitted to hospital*	12 (5.3)		
*Shelter*	32 (14.3)		
*Discharge against medical advice*	13 (5.8)		

Note: ^a^SD refers to standard deviation.

### Pattern of HNF injuries

**Figure 1:**
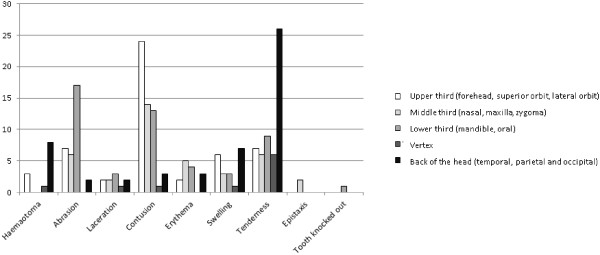
The types and locations of IPV-related head and maxillofacial injuries in abused women (n = 223).

HNF injuries were the most frequently reported injuries (n = 173, 77.6%) in women. Figure 1 shows the anatomical regions and types of head and maxillofacial injuries. The majority of injuries affected the soft tissue; for example, contusion (n = 109, 28%), abrasion (n = 62, 16%), swelling (n = 39, 10%), erythema (n = 25, 7%), hematoma (n = 23, 6%), laceration (n = 17, 5%), epistaxis (n = 2, 0.5%) and knocking-out of a tooth (n = 2, 0.5%). No fracture was found among the HNF injuries. The most common aetiology of the injury was punching with a fist (n = 133, 60.2%), followed by slapping (n = 39, 17.6%), pushing or shoving or grabbing (n = 34, 15.4%), weapon use (n = 25, 11.8%), kicking (n = 26, 11.8%), arm or hair twisting (n = 19, 8.6%), slamming against a wall (n = 17, 7.7%), choking (n = 16, 7.2%), throwing things (n = 10, 4.5%) and human bites (n =3, 1.4%). In 40.2% of cases, more than one cause of abuse was reported. Weapons used included blunt objects (81.8%), such as wooden sticks, chairs, clothes hangers, slippers and umbrellas, and penetrating objects (18.2%), such as knives, scissors and needles.

Chi-squared tests were performed to understand the relationships of injury aetiology with injuries of different parts of the head and maxillofacial region. The results showed that injuries to the upper third of the face and the back of the head were significantly associated with punching with a fist (*X*^2^ = 6.54, *p* = .01; *X*^2^ = 4.94, *p =* .03), while injuries to the middle third of the face were significantly associated with slapping (*X*^2^ = 4.2, *p =* .04).

Table 2 shows the structured multiphase logistic regression models that examined the risk factors of HNF injuries. The models indicated no significant risk factor in phases I and II. However, in phase III, punching with a fist was found to be significantly associated with HNF injuries (OR = 2.4, 95% CI = 1.2, 5.1; *p* = .013). Although kicking is another significant aetiology of injuries, it was found unlikely to yield HNF injuries (OR = .3, 95% CI = .1, .7; *p* = .01).

**Table 2:** Structured multiphase logistic regression model of demographics, causes of injury and injury episodes associated with head, neck, and face injuries (n = 199)

	**Head, neck, and face injuries**		
	**OR**^**a**^	**95% CI**^**b**^	***p***
**Phase I (Nagelkerke R square = 0.6%, Hosmer and Lemeshow Test** ***p =*** **.92)**			
**Age**			.856
*≤ 29*	1.0		
*30-49*	1.2	(.5, 2.8)	
*≥ 50*	.9	(.3, 2.7)	
**Race**			.989
*Chinese*	1.0		
*Non-Chinese*	1.0	(.4, 2.7)	
**Phase II (Nagelkerke R square = 1.2%, Hosmer and Lemeshow Test** ***p =*** **1.0)**			
**Relationship with perpetrator**			.604
*Married spouse*	1.0		
*Cohabitant*	1.6	(.6, 4.6)	
*Boyfriend but non-cohabitant*	.3	(0, 4.8)	
*Ex-husband / ex-boyfriend*	1.5	(.2, 13.0)	
**Phase III (Nagelkerke R square = 15.1%, Hosmer and Lemeshow Test** ***p =*** **.28**			
**Episode of abuse**			.8
*1*^*st*^	1.0		
*2*^*nd*^	1.1	(.3, 4.1)	
*Multiple*	.7	(.3, 2.0)	
*Undisclosed*	.6	(.2, 2.3)	
**Causes of injury**^**c**^			
*Punching with a fist*	2.4	(1.2, 5.1)	.013*
*Slapping*	2.6	(.8, 8.3)	.097
*Pushing, shoving or grabbing*	1.0	(.4, 2.7)	.930
*Weapon use*	.8	(.4, 1.9)	.688
*Kicking*	.3	(.1, .7)	.01*
*Arm or hair twisting*	.5	(.2, 1.6)	.248
*Slamming against a wall*	2.1	(.4, 10.4)	.381
*Throwing things*	1.2	(.2, 6.4)	.835
**Admission time**			.429
*Day-time*	1.0		
*Night-time*	0.8	(.4, 1.5)	

Note: ^a^OR refers to odds ratio; ^b^CI refers to confidence interval; Causes of injury; ^c^The comparators are “no punching with a fist”, “no slapping”, “no pushing, shoving or grabbing”, “no weapon use”, “no kicking”, “no arm or hair twisting”, “no slamming against a wall”, “no throwing things” respectively; *p < 0.05; Tolerance > 0.9 and VIF < 1.1.

### Multiple injuries

Of the abused women, 53.4% who had HNF injuries also suffered from chest, abdominal, back, pelvic or extremities injuries. Table 3 shows the structured multiphase logistic regression models that indicate the risk factors of multiple injuries involving the head. It was found that the relationship with the perpetrator was the significant factor of multiple injuries in abused women in phase II (*p* = .041). Women who were cohabitating with the perpetrator were at much greater risk of having multiple injuries (OR = 3.3, 95% CI = 1.4, 7.8). Having a former partner as the perpetrator was also found to be associated with a greater risk of multiple injuries (OR = 2.1, 95% CI = .4, 11.9). Punching with a fist was also found to be a significant risk factor in having multiple injuries (OR = 4.0, 95% CI = 2.1, 7.5, *p* < .001).

**Table 3:** Structured multiphase logistic regression model of demographics, causes of injury and injury episodes associated with multiple injuries (n = 199)

	**Multiple injuries**		
	**OR**^**a**^	**95% CI**^**b**^	***p***
**Phase I (Nagelkerke R square = 3.3%, Hosmer and Lemeshow Test** ***p =*** **.99)**			
**Age**			.418
*≤ 29*	1.0		
*30-49*	.6	(.3, 1.3)	
*≥ 50*	.6	(.2, 1.5)	
**Race**			.098
*Chinese*	1.0		
*Non-Chinese*	2.1	(.9, 4.9)	
**Phase II (Nagelkerke R square = 5.5%, Hosmer and Lemeshow Test** ***p =*** **1.0)**			
**Relationship with perpetrator**			.041*
*Married spouse*	1.0		
*Cohabitant*	3.3	(1.4, 7.8)	
*Boyfriend but non-cohabitant*	1.1	(.1, 17.0)	
*Ex-husband / ex-boyfriend*	2.1	(.4, 11.9)	
**Phase III (Nagelkerke R square = 18.2%, Hosmer and Lemeshow Test** ***p =*** **.23)**			
**Episode of abuse**			.894
*1*^*st*^	1.0		
*2*^*nd*^	1.5	(.5, 4.1)	
*Multiple*	1.2	(.5, 2.8)	
*Undisclosed*	1.3	(.5, 3.9)	
**Causes of injury**^**c**^			
*Punching with a fist*	4.0	(2.1, 7.5)	<0.001*
*Slapping*	1.4	(.6, 3.2)	.377
*Pushing, shoving or grabbing*	1.6	(.7, 3.7)	.251
*Weapon use*	1.2	(.6, 2.5)	.545
*Kicking*	1.2	(.5, 3.1)	.670
*Arm or hair twisting*	1.0	(.3, 2.9)	.976
*Slamming against a wall*	1.2	(.4, 3.7)	.749
*Throwing things*	3.1	(.6, 15.0)	.167
**Admission time**			.747
*Day-time*	1.0		
*Night-time*	1.1	(.6, 2.0)	

Note: ^a^OR refers to odds ratio; ^b^CI refers to confidence interval; Causes of injury; ^c^The comparators are “no punching with a fist”, “no slapping”, “no pushing, shoving or grabbing”, “no weapon use”, “no kicking”, “no arm or hair twisting”, “no slamming against a wall”, “no throwing things” respectively; *p < 0.05; Tolerance > 0.9 and VIF < 1.1.

## Discussion

This is the first study that examined HNF injuries in abused Chinese women presenting to AEDs. HNF injuries (77.6%) were the most frequently reported injuries among abused women in this study, which is consistent with previous findings in Caucasian women presenting to AEDs [8,28]–[30]. Also, it was found that HNF injuries mostly resulted from punching with a fist (60.2%).

Considering the head in terms of five areas, namely the upper third, middle third, and lower third of the face, the vertex of the head, and the back part of the head, the findings showed that the back part of the head was the most frequently affected region. In addition, women who had injuries to the back part of the head had mostly been punched with a fist. Repeated attacks with the fist at any one site can generate enough force to cause fractures [31]. In our sample of IPV women, we fortunately did not observe any fracture to the back part of the head, which may be because the punches were few or exerted with less force. However, haematomas were commonly found on the back part of the head, mainly in the parietal and occipital regions. All the women with haematomas had soft tissue injuries rather than subdural or epidural hematomas, as identified from X-rays or CT scans, which are much more severe injuries. The relationship between soft tissue injuries and severity of trauma has been examined and found that the formation of a haematoma indicated moderate trauma, while massive haematoma indicated severe trauma [32]. With moderate to severe blunt force, it may be possible to cause micro-structural tissue damage inside the parietal and occipital lobes of the brain, which may affect the somatosensory and visual functioning. Unfortunately, this micro-structural tissue damage may not be easily recognized by current routine investigations (i.e. X-rays or CT scans) for head injuries at AEDs. Therefore, the exact level of brain damage cannot be determined. Magnetic resonance imaging (MRI) or functional MRI may provide further evidence on the neuroanatomical impact in abused women.

The lower third of the facial region was the second most frequently affected region among all HNF injuries. This finding is inconsistent with the previous studies conducted in clinical and community samples in Western countries, which found that injuries were most commonly found in the middle third of the facial region [30,33]. Moreover, neither punching with a fist nor slapping were significant aetiologies of lower third maxillofacial injuries. It may be possible that the lower third maxillofacial injuries is the consequence of either a fall or knock down followed by punches or slaps.

IPV-related injuries occurred repeatedly among abused women presenting to AED. Around 70% of women reported that the abuse was not the first episode. This is consistent with the findings in many studies conducted in abused women [8,34,35]. An important finding to be noted is that almost 80% of the women with IPV-related physical injuries returned home after AED visits. It is likely that these women will return to their abusive partners for a variety of reasons such as financial dependence, emotional dependence, and protection of their children [36,37]. Although women’s choice of staying in or leaving their abusive relationships should be respected, the stay can be dangerous especially for women with repeated episodes of IPV-related injuries. Therefore, AED physicians and nurses play a vital role in early intervention and empowering women with personal safety issues.

More than half of the women reported HNF injuries combined with other injuries of different body regions; thus, we repeated the structured multiphase logistic regression analysis with multiple injuries as the dependent variable. The woman’s relationship with the perpetrator was found to be a significant factor associated with multiple injuries. In particular, unmarried cohabitating women were 3.3 times more at risk of having multiple injuries than married cohabiting women. A population-based study also found that cohabiting couples had more violent relationships than married couples [38]. It may be possible that cohabiting couples with lower levels of violence tend to move from cohabitation to marriage, whereas couples with higher levels of violence tend to be trapped in violent cohabiting relationships. In addition, women who had former partners as perpetrators were much more at risk of having multiple injuries. It is also possible that separation may be involved in and initiated the abuse. Indeed, previous studies found that separation after a cohabiting relationship or marriage was associated with a higher risk of physical abuse and homicide [21,39,40].

Several limitations were found in this study. Firstly, this study relies on secondary clinical data from medical charts with missing values ranging from 0% to 32%. The accuracy and integrity of the data abstracted remained a shortcoming. However, the hospitals chosen for data collection were two main hospitals in the region with the highest reported number of abused women. Also, the hospitals have kept completed medical charts and IPV assessment records. Hence, incompleteness and inadequate information were kept to a minimum. In addition, efforts have been made to minimize misinterpretation of information from medical charts by using two independent reviewers and consensus meetings. Furthermore, the standard chart abstraction sheet specially designed in this study aimed to facilitate data collection and minimize missing data. Secondly, little information regarding the problem, conflict or dispute that initiated the abuse could be found from the clinicians’ notes. A prospective study to investigate the conflicts, dispute and communication patterns immediately before the episode of abuse would be essential to develop warning signs of physical abuse. Thirdly, data collected were based on physical examinations and women’s self-report of injuries. It is possible that the women neglected to mention what they regard as minor injuries. Therefore, underestimation of physical injuries in abused women may occur. In addition, the findings of the present study may not be generalizable to women in the community because the physical injuries reported at AEDs tend to be those that are much more severe. It is possible that replication of this study by using a community sample would yield more non-HNF injuries among abused women. Future study to investigate the pattern of physical injuries in the primary care setting is recommended.

### Implications for research

Although HNF injuries are commonly found in abused women presenting to AEDs, limited studies investigated the long term effect on physical and mental health of women after HNF injuries. Some cross-sectional studies assessed for cognitive functioning (such as memory, attention, executive functions and learning) among abused women [28,41,42]. Therefore, future research in longitudinal design is necessary to test for the temporal relationship between cognitive functioning and HNF injuries in abused women experiencing abuse and violence.

Apart from obtaining archival data from AEDs, screening of HNF injuries in community-dwelling women is recommended. It can provide a better understanding of physical injuries among abused Chinese women. We anticipated that having a community sample would yield more non-HNF injuries among abused women.

### Implications for practice and/or policy

This is the first study to document detailed information on HNF injuries in abused Chinese women. It provides descriptions of the relationships between injuries and its aetiology. Therefore, the present study contributed valuable information regarding the features of physical injuries experienced by abused Chinese women.

The study collected data from two local hospitals with a standard protocol for IPV assessment. This provided evidence that direct questioning for IPV-related patients with physical injuries at AEDs, and the history and details of the mechanism of injuries, are important for early identification in order to prevent further abuse. Although it is the women’s choice to stay in or leave abusive relationships, women sometimes underestimate the danger and risks of situations [43]. It is recommended that health care professionals should increase the screening of women with physical injuries to better identify survivors and promote awareness of IPV dangers. The U.S. Preventive Services Task Force recently released a report and recommended to do IPV screening to asymptomatic women (women who do not have signs or symptoms of abuse) of reproductive age, elderly and vulnerable adults [44].

One important finding in this study is that cohabiting and separated women were more likely to have multiple injuries than those who were married. Therefore, when cohabiting or separated women are presented to AEDs, they should be given special attention regarding early identification, intervention, and information of abuse. The extra attention may keep them safe and protect them from further abuse and repeated injuries.

## Conclusions

IPV has been recognized as an important issue among health care professionals. The present study helps understand the pattern of HNF injuries, including anatomical regions, type, severity and aetiology in abused Chinese women. HNF injuries remain to be the most common injuries reported in abused Chinese women presenting to AEDs. Comprehensive data analysis found that punching with the fist was the most common aetiology and had a significant association with upper third maxillofacial injuries and injuries to the back of the head. Cohabiting and separated women were much more likely to have multiple injuries. As Chinese abused women were typically reluctant to disclose abuse because of fear and shame, these findings are essential to help clinicians improve diagnosis for women.

## Competing interests

All authors declare that they have no competing interests. The present study participants were also not in the authors’ previously published work.

## Authors' contributions

JW carried out data collection, data analysis and drafted the manuscript. AC participated in data collection. DF participated in the design of the study, data analysis and helped draft the manuscript. KSW and CLL participated in data collection and coordination. CWK conceived of the study and coordination. All authors read and approved the final manuscript.

## Pre-publication history

The pre-publication history for this paper can be accessed here:

http://www.biomedcentral.com/1472-6874/14/6/prepub
